# Immigration from Africa to the United States: key insights from recent research

**DOI:** 10.3389/fsoc.2023.1171818

**Published:** 2023-06-08

**Authors:** Mamadi Corra

**Affiliations:** Department of Sociology, East Carolina University, Greenville, NC, United States

**Keywords:** African immigrants, immigration from Africa, black immigrants, race, immigration

## Abstract

Immigration from Africa to the United States has increased dramatically in the past three decades. This paper summarizes recent findings on the growth of African immigration to the United States in recent years. In doing so, it highlights shifting sociodemographic profiles of these “new African Americans” or “new Americans,” profiling the increasing diversity, yet also racialized portrait of this group. Key patterns of immigration shown include the changing racial and gender composition of immigrants, as well as rising immigration from a wider range of African countries. Some key theoretical and practical implications are outlined.

## Introduction

Today, African immigrants constitute a growing and increasingly visible component of the U.S. population (Thomas, 2014; Elo et al., 2015; Hamilton, 2020; Corra, 2022; Tamir, 2022; Tamir and Anderson, 2022). In the following pages, I review recent patterns and trends of the growth of African immigration to the United States (Corra, 2022). And, in doing so, I highlight some notable emerging demographic processes that continue to follow from this growth, as well as key questions that can be inferred from them.

Notably, an objective of the “Insights in Migration and Society: 2022” series is for articles that summarize key insights and developments in an area within the scope of Migration and Society, while also enabling authors to *highlight their current research*. To that end, the scope of this paper is to profile findings from a recent analysis of U.S. Census data (Corra, 2022). And, in so doing, the goals of the review presented in the following pages are threefold. First, I outline recent patterns and trends shown to be associated with the flow of African immigration to the United States (Elo et al., 2015; Corra, 2022; Tamir, 2022; Tamir and Anderson, 2022). Second, I offer a summary of shifts shown in the demographic composition of the U.S. African immigrant population itself (Elo et al., 2015; Tamir, 2022; Tamir and Anderson, 2022), and the impact of such shifts on U.S. population subgroups (Corra, 2022). In doing so, I offer key research findings shown to be associated with the African immigrant population (Elo et al., 2015; Hamilton, 2020; Corra, 2022). Finally, I seek to offer some theoretical and practical insights that can be inferred from all of these.

## Dramatic increase in the flow of immigrants in the past three decades

**Figure 1:**
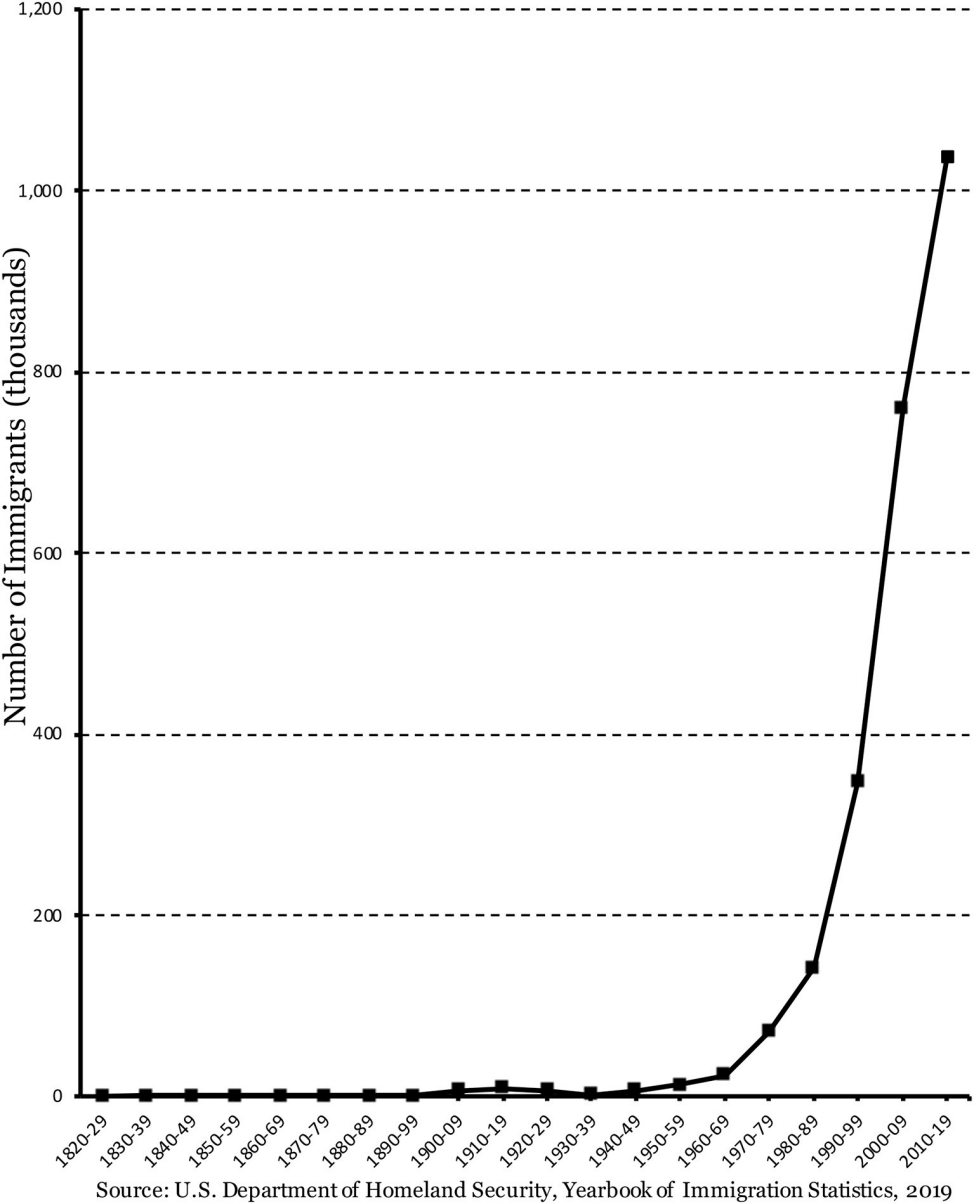
Africans obtaining legal permanent residence status, by year, 1820–2019, in thousands. Source: U.S. Department of Homeland Security (DHS) (2019).

Figure 1 presents data on African immigration to the United States from the 1820s to the 2010s. From the U.S. Department of Homeland Security (DHS) (2019), data presented in that figure is representative of the total number of Africans who have obtained legal permanent residence (LPR) status in the United States, by decade, beginning with the 1820s to the 2010s.^1^ More specifically, this graph is a cumulative depiction of which for each decade on the *x*-axis, the value on the *y*-axis represents the total number of Africa-born persons who have ever received LPR status.

As can be seen in Figure 1, “voluntary immigration”^2^ from Africa to the United States, at least in large numbers, is a very recent phenomenon (Corra, 2022). For example, data presented in that figure shows that African immigration to the United States was relatively small until the decade of the 1900s. It continued to remain so for the next few decades but began to take off in the 1960s and 1970s, and noticeably starts to accelerate thereafter. And, by the decades of the 2000s and 2010s, this pattern of rapid growth is shown to be distinctly evident.

Consider the values presented in Figure 1, for example, in terms of percent growth. Notably, of the total number of Africans that were granted legal permanent residency in the United States between the 1820s and 2010s, the proportion of African immigrants did not reach the 1% mark until the decade of the 1960s. In other words, about 99% of Africa-born persons who have ever received LPR status in the United States acquired this status after the decade of the 1960s. This pattern of noticeable, but negligible growth remained until the 1990s, when the percent of Africa-born persons who have ever received LPR status is in the double digits, about 15%. And by the 2000s, the percent of Africa-born persons who have ever received LPR status more than doubles to about 33% of the total. By the decade of the 2010s (2010–2019), this percentage increases appreciably to about 40%. Taken together, close to 90% (about 88%) of Africa-born persons who have ever received LPR status in the United States between the 1820s and 2010s acquired that status in the last three decades (1990s, 2000s, and 2010s).

In summary, the data presented in Figure 1 shows an appreciable increase in the total number of African immigrants to the United States in recent years, noticeably in the 1990s and accelerating in the 2000s and 2010s.^3^ Several factors are cited in the literature for this dramatic increase, including changes in U.S. immigration policy, economic and political instability in some African countries (Kollehlon and Eule, 2003; Jasso, 2011; Thomas, 2011; Logan and Thomas, 2012). The Immigration Act of 1990, for example, introduced a Diversity Visa Lottery Program designed to increase the number of immigrants from countries underrepresented in the United States. Yet, that program also required attainment of certain basic educational and work-related skills of immigrants premigration. And research shows that many recent immigrants from Africa acquired legal permanent residence status under this program (Corra, 2022). Accordingly, this program has led to the increase of the number of African immigrants admitted on the basis of job skills, thus providing new avenues for highly skilled/educated African immigrants to immigrate to the United States (Lobo, 2001; McCabe, 2011; Thomas, 2011; Hamilton, 2020).

It is in this light that some scholars have observed that, collectively, immigration from Africa in the past two decades has been the largest flow of Africans to the U.S. since the trans-Atlantic slave trade (Roberts, 2005; Konadu-Agyemang and Takyi, 2006; Anderson and López, 2018). As an example, data presented by Kent (2007) showed the annual number of African arrivals in the United States to have been close to 60,000 between 2000 and 2005. By contrast, an estimated 460 African immigrants arrived from Africa to the United States annually between the years 1861 and 1961 (Konadu-Agyemang and Takyi, 2006).

When it comes to source countries, however, recent analysis of U.S. Census data (Corra, 2022) suggests that two patterns of African immigration to the United States are evident. First, a limited number of long-standing sending countries continue to be the source of a sizeable proportion of African immigration to the United States. Analyzing 1980, 1990, 2000, 2010, and 2019 U.S. Census data, for example, Corra (2022) reports that 10 “top sending” countries^4^ represented about 71%, 76%, 71%, 69%, and 70%, respectively, of the total number of African immigrants in the United States. As an example, consider Egypt as a source country. That country alone is shown to have accounted for about 20%, 19%, 13%, 9%, and 9%, respectively, of the total number of African immigrants in the United States in 1980, 1990, 2000, 2010, and 2019. Similarly, Nigeria alone is shown to have accounted for more than one out of every 10 African immigrants in the United States in those five time periods: about 14%, 14%, 16%, 14%, and 16%, respectively (Corra, 2022). Taken together, Corra (2022) reports that, with only a few exceptions, Nigeria, along with Ethiopia, Egypt, South Africa, Ghana, and Morocco, have consistently been among the top 10 countries of African immigrants in the United States in the past few decades. Corra calls this a “concentrated” flow/representation of immigration from a small number of African countries.

Yet, a second emerging trend in the flow of African immigration to the United States is also shown to be evident. This is the rising immigration from a wider range of African countries, i.e., increasing diversification of source countries (Corra, 2022). According to Corra (2022), the flow of African immigrants from a limited number of long-term sending countries has been on the decline (see the example of Egypt above). In fact, Corra's analysis shows that top 10 sending countries have changed over the years, with some countries reaching that threshold (and some dropping below) as time progressed. This is argued to be an indication of growing “diversity” in the flow of African immigrants to the United States in recent years (Corra, 2022).

As an example, consider variations in legal admission status. The U.S. Immigration and Nationality Act (INA) provides several broad classes of admission for foreign nationals to gain legal permanent residency (LPR) status in the United States. Those classified as immediate relatives of U.S. citizens include spouses, children, and parents of U.S. citizens age 21 and older. Those admitted based on family-based preferences include relatives/Family members not included in the immediate relatives class of admission, e.g., married, or unmarried adult sons/daughters of U.S. citizens, brothers/sisters of such citizens, etc. Specific subcategories in the family-based preferences include “Family First Preference” (Unmarried sons/daughters, over the age of 21, of US citizens), “Family Second Preference” (Spouses and unmarried children of Permanent Residents), “Family Third Preference” (Married sons/daughters of US citizens), and “Family Fourth Preference” (Brothers and sisters of US citizens). Admissions based on employment are given to those seeking to provide needed skills in the U.S. workforce or invest in new U.S. jobs, along with their dependents. Refuge is granted to two sets of immigrants who have been persecuted or have a “well-founded” fear of persecution, refugees and asylees. Refugees are those admitted outside the United States with their immediate relatives, while asylum is given to those seeking refuge, but are already inside the United States, and their immediate relatives. Finally, those gaining LPR based on the Diversity program come from countries with relatively low levels of immigration to the United States. (For a fuller description of these classifications, see descriptions at the DHS site https://www.dhs.gov/immigration-statistics/lawful-permanent-residents/ImmigrantCOA).

The rising immigration from a wider range of African countries is shown to be associated with notable variations in legal admission status. Analyzing data for the years 1996, 1998–2019, as reported by the U.S. Department of Homeland Security, Yearbook of Immigration Statistics, Corra (2022), for example, shows that well over 80% of immigrants from Somalia were granted legal status under the Refugee and Asylee category, more than 50% of immigrants from the Democratic Republic of Congo and Sudan, and about 47% of immigrants from Tanzania acquired legal status under this admission category. By contrast, more than 40% of immigrants from South Africa acquired legal status under employment-based preferences. Whereas almost all other countries show percentages in the single digits as employment-based preference admittees. Importantly, analysis of recent data (Corra, 2022) also shows that immigrants from many African countries were admitted under the diversity category (over 20% for most top sending countries, and well over 30% for several of these). Here, I note that theory and research suggest that the type of U.S. visa immigrants hold impacts their incorporation into U.S. society (Jasso et al., 2000; Jasso, 2004),^5^ and recent analysis of U.S. Census data (Corra, 2022) provides strong support for this proposition.

Moreover, shifts in regional representation are also shown to be evident, with African immigrants from Northern and Southern Africa shown to be decreasing proportionately, while immigrants from Central, East, and West Africa increasing or holding steady (for details, see Corra, 2022, Chapter 4). Immigrants from Central Africa, for example, are shown to have represented about 3% of the total number of African immigrants in the United States in 1980. By 2019, however, the percentage of immigrants from this region is shown to have increased to about 9%, a growth representing a twofold increase. By contrast, the percent of immigrants from North Africa was as high as 39 in 1980. By 2019, however, that percentage had dropped dramatically to about eighteen, a more than one half decline (Corra, 2022).

## A pattern of changing racial/ethnic composition of immigrants

**Figure 2:**
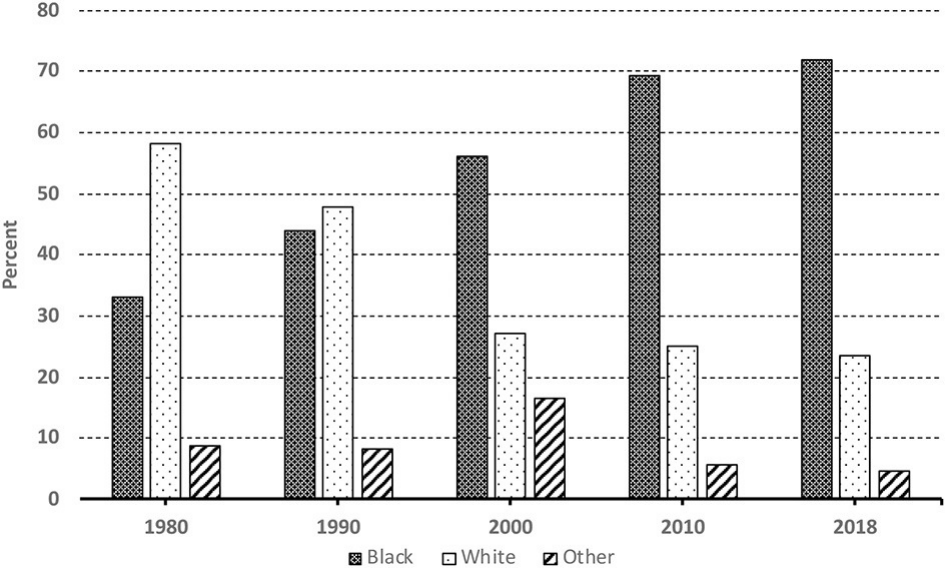
Immigrants from Africa to the U.S. 1980–2018 by race.

Recent research suggests that one clear pattern directly flowing from the current growth of African immigration to the United States is the changing racial/ethnic composition of the U.S. African immigrant population (Thomas, 2014; Corra, 2022). To illustrate, consider data displayed in Figure 2. Representative of samples from waives of the 1980, 1990, and 2000 decennial U.S. Censuses and the 2010 and 2018 American Community Surveys (ACS), as represented in the Integrated Public Use Microdata Series (IPUMS) (Ruggles et al., 2020),^6^ Figure 2 depicts the racial composition of the U.S. African immigrant population, for the time periods 1980, 1990, 2000, 2010, and 2018.

As displayed in Figure 2, the trend shown is a growing proportion of self-identified “Black” African immigrants, on one hand, and a decreasing proportion of self-identified “White” immigrants, on the other hand (for a similar pattern, see Borch and Corra, 2010; Elo et al., 2015; Tamir and Anderson, 2022). For example, the data presented in Figure 2 indicates that about 33% of African immigrants in the United States self-identified as Black in 1980. That percentage increased to about 44% by 1990, 56% by 2000, 69% by 2010, and finally 72% by 2018. By contrast, the percent of African immigrants identifying as White begins at almost 60% in 1980—about 58%. It declines appreciably to 48% by 1990, declines again dramatically to 27% by 2000, to 25% by 2010 and ends up down to about 24% by 2018 (see Corra, 2022, Chapter 2, for a more detailed discussion).

In short, until very recently, voluntary immigration (see text footnote 2 above) from Africa to the United States has been disproportionately those who identified themselves as White. More recent immigration from Africa, by contrast, has increasingly included Black Africans (Borch and Corra, 2010; Elo et al., 2015).

More generally, while the presence of immigrants from most African countries has been on the rise, a notable point to make is that the recent growth has been greater from some regions and countries than others, and thus the changing proportionate patterns represented in Figure 2. In other words, immigration from most African countries has been on the rise, but the recent dramatic increase is more pronounced among immigrants from Sub-Saharan Africa (Elo et al., 2015; Corra, 2022; Tamir, 2022; Tamir and Anderson, 2022).

Yet, one perspective on the foregoing shift in the racial composition of African immigrants to the U.S., is that a key reason why the percentage of White Africans was high in the past is due to the exclusion of “Whites” from Egypt and South Africa from the national origin quotas used to restrict non-European immigration to the U.S. in the early 1900s (Gordon, 1998; Thomas, 2014).^7^ This meant a less restrictive immigration of “Whites” from these two countries. In 1965, however, amendments to the Immigration and Naturalization Act abolished the old national origins system, ushering in a new era that emphasized preference for employment and family-based immigration. Thus, a possible force behind the shift in the racial composition of immigrants may be shifts in immigration policy (see text footnote 7).

The recent decline in White African immigration, however, remains notable, especially given the fact that immigrants from these two countries still predominantly report high percentages of self-identified “Whites” (see Dodoo and Takyi, 2002; Kollehlon and Eule, 2003; Borch and Corra, 2010; Corra, 2022). Furthermore, while immigration of Africans of Arab descent who come predominantly from North and East Africa is on the rise (see the paragraph to immediately follow), recent African immigration to the U.S. has clearly shifted to those predominantly coming from Subsaharan Africa (Elo et al., 2015; Anderson and López, 2018; Tamir, 2022; Tamir and Anderson, 2022).

Moreover, a key variable associated with recent waves of immigrants from Africa is the increasing inclusion of individuals of Arab ethnic origins that come mainly from North and East Africa. According to one estimate, between 1990 and 2000, Arab immigration from Egypt, for example, increased by 82% (De la Cruz and Brittingham, 2003). Importantly, 80% of Arab immigrants identify themselves as “White” (De la Cruz and Brittingham, 2003).

## Resulting changes in the composition of the foreign-born and U.S. Black populations

Taken together, two resulting sociodemographic processes already underway in the U.S. are notable. First, today, African immigrants constitute a rising and increasingly visible component of the U.S. Black population (Logan and Deane, 2003; Shaw-Taylor and Tuch, 2007; Capps et al., 2012; Corra, 2022). Second, African immigrants also comprise a growing proportion of the U.S. foreign-born Black population.^8^

A recent Pew Research Center analysis (Anderson and López, 2018), for example, revealed that, between 2000 and 2013, the number of black African immigrants living in the U.S. rose by about 137%, from 574,000 to 1.4 million. And that Africans made up 36% of the total foreign-born black population in 2013, up from about 24% in 2000 and just 7% in 1980 (Corra, 2022). The title of a Pew Research Center analysis released just last year (January 2022), “The Caribbean is the largest origin source of Black immigrants, but fastest growth is among African immigrants,” is decidedly direct. That report notes that Black immigrants from Africa have been the primary driver for much of the overall recent growth in the U.S. Black immigrant population. The authors of that report (Tamir and Anderson, 2022) note that, between 2000 and 2019, the Black African immigrant population grew 246%, from roughly 600,000 to 2.0 million. And that Black African immigrants now make up 42% of the overall foreign-born Black population, almost double of this percentage in 2000 when that share was about 23%.

As Corra (2022) perceptively observes, these “new African Americans” (Millman, 1997, 172) or “new Americans” (Barone, 2001) are adding to the increasing diversity and racial/ethnic transformation that U.S. society is currently experiencing (Shaw-Taylor and Tuch, 2007; Anderson, 2015; Tamir, 2022). For example, scholars and commentators alike have observed that the new flow of African immigration to the United States is further contributing to the remaking of the U.S. Black population in fundamentally different ways (Shaw-Taylor and Tuch, 2007; Hamilton, 2019, 2020). A 2015 Pew Research Center report (Anderson, 2015) projects that, by 2060, 16.5% of the U.S. black population will be foreign-born, and a recent analysis of U.S. Census data (Corra, 2022) suggests that Black African immigrants will constitute a sizeable portion of this projected growth.

An important question that inevitably flows from the foregoing is how these immigrants are adapting into the social and economic fabric of their new country? (Corra, 2022). And importantly, what theoretical insights can be gained by investigating this group of immigrants who are mostly Black? (See Tamir, 2022; Tamir and Anderson, 2022). According to Corra (2022), for African immigrants, answers to these questions are confounded by the fact that they are both immigrants and for many, black^9^—two socially significant variables shown to influence immigrant adaptation in the United States.^10^ According to Dodoo and Takyi (2002): “The condition of Africans in the diaspora proffers insight into not just their adaptation to their new countries, but also the nature of racial stratification at their destinations” (p. 913).

## A trend of changing gender composition of immigrants toward parity

A second clear pattern directly flowing from the recent growth of African immigration to the United States is the shifting gender composition of the U.S. African immigrant population toward parity: a shift from majority male to parity—half of African immigrants living in the United States today are male and half are female.

**Figure 3:**
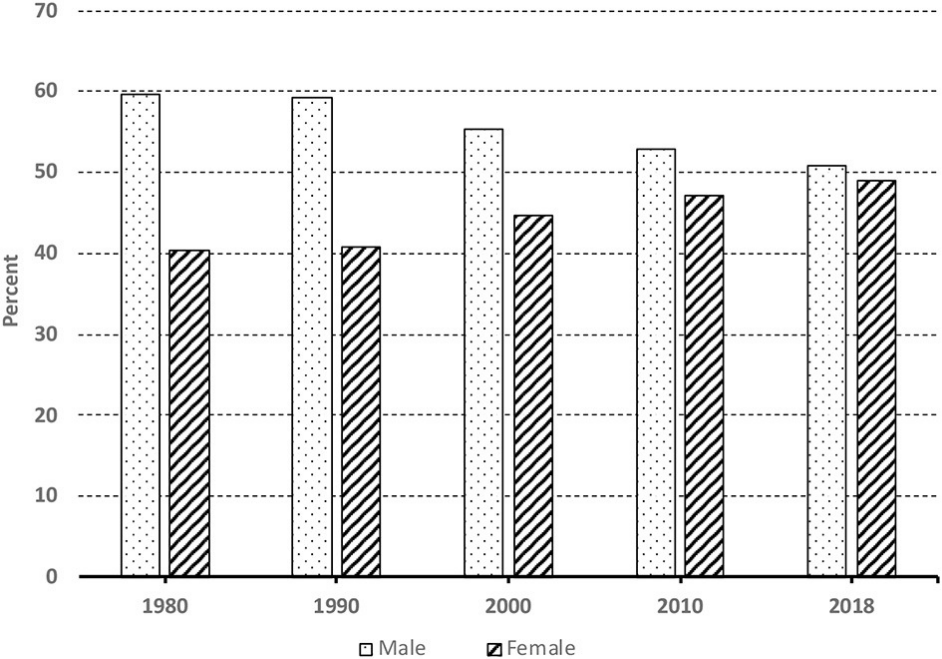
Immigrants from Africa to the U.S. 1980–2018 by gender.

To illustrate, consider data displayed in Figure 3. Representative of samples from waives of the 1980, 1990, and 2000 decennial U.S. Censuses and the 2010 and 2018 American Community Surveys (ACS), as represented in the Integrated Public Use Microdata Series (IPUMS) (Ruggles et al., 2020), Figure 3 depicts the gender composition of the U.S. African immigrant population, for the time periods 1980, 1990, 2000, 2010, and 2018. As can be seen in that graph, in 1980, three out of every five African immigrants living in the United States were male. By 2018, that number had declined to one out of every two–exactly half of the total African immigrant population.

Importantly, the significance of gender in status attainment in the United States is well-documented in the sociological literature.^11^ Yet, according to Corra and Kimuna: “The experience of Black female immigrants to the United States has been ignored in discussions of economic outcomes, mainly because they have been traditionally viewed as “dependents,” moving as wives, mothers or daughters of male migrants” (p. 1032). Current waves of female immigrants from Africa to the United States (Corra, 2022), however, suggest the need for a re-examination. And recent analysis of U.S. Census data (Corra, 2022) illustrates that the increasing presence of female immigrants (especially black/African female immigrants) may indeed be changing the size, demographic composition and dynamics of the U.S. labor market in fundamental ways (see below).

Key issues of research here are whether or not the socioeconomic trajectories of male and female immigrants from Africa continue to be markedly different from one another. And if so, to what extend the patterns mirror those shown for male and female natives, as well as those shown for other immigrant groups. And related questions include: How have these patterns changed in the past several decades? How might they change in the next few decades? And what do answers to these questions suggest about the state of theoretical understandings of the salience of gender for status attainment in contemporary America?

## Some key recent empirical findings

In examining the status of African immigrants in the United States, at least three levels of analyses are possible. First, groups of African immigrants can be compared with one another (e.g., Dodoo and Takyi, 2002; Borch and Corra, 2010; Thomas, 2014; Hamilton, 2020; Corra, 2022). Second, African immigrants can be compared with other immigrant groups (e.g., Dodoo, 1997; Model, 2008; Corra and Kimuna, 2009; Corra and Borch, 2014; Kusow et al., 2016, 2018; Hamilton, 2020; Corra, 2022). Finally, African immigrants can be compared with native-born groups (e.g., Dodoo, 1997; Model, 2008; Corra and Kimuna, 2009; Corra and Borch, 2014; Kusow et al., 2016; Hamilton, 2020; Corra, 2022). Here, I briefly note recent key findings as they pertain to each of these three.

### African immigrants compared with one another

Comparing Africans with one another, several points are worth noting here. First, it may be noted that the salience of race for socioeconomic attainment in contemporary America is well-documented in the sociological literature (Farley, 1984; Burstein, 1985; Tomaskovic-Devey, 1993; Cancio et al., 1996; Borch and Corra, 2010; Thomas, 2014; Corra, 2022). And when it comes to African immigrants, recent evidence shows race to be a key distinguishing characteristic (Dodoo and Takyi, 2002; Borch and Corra, 2010; Thomas, 2014; Hamilton, 2020; Corra, 2022). Black immigrants from Africa to the United States, for example, are consistently shown to fair worse in the U.S. labor market than their white African counterparts (Dodoo and Takyi, 2002; Borch and Corra, 2010; Thomas, 2014; Hamilton, 2020; Corra, 2022).

An early example is Dodoo and Takyi's (2002) study. In exploring race differences in earnings between Black and White African immigrants in the U.S., Dodoo and Takyi (2002) report “sizeable differences among immigrants who have relatively similar human capital” (p. 913). They find that “Whites have annual earnings 80% higher than their Black counterparts, and the gap in hourly wage is almost 48%” (p. 913). Notably, Dodoo and Takyi (2002) report that “more than half (53%) of the race difference in wages remains unexplained by earnings-related attributes such as education, occupation, and hours worked” (p. 913). Borch and Corra (2010) subsequent analysis of three decades of Census data (1980, 1990, and 2000) also found race effects, with the gap in earnings between Black and White male immigrants shown to have especially widened over time.

Corra (2022) more recent analysis shows more nuanced findings that are shown to be gendered. What he calls the “gendered significance of race,” Corra's analysis shows that, relative to white African male migrants, black African male migrants have poorer socioeconomic outcomes than black African female migrants do (relative to white African female migrants).

Gender is also shown to be an independently salient variable. Consistent with the extent literature, on average, a variety of socioeconomic measures for African immigrant men are shown to be noticeably more favorable than those shown for women. For example, notable disparities in self-employment between men and women are documented, with men reporting self-employment rates that are two to three times higher than those reported by women (see Corra, 2022). Moreover, female African immigrants are consistently shown to hold occupations with lower prestige than men, and to earn less (see Borch and Corra, 2010; Corra, 2022).

Finally, country-level differences are shown to exist. The U.S. labor market apparently favors immigrants from some African countries than others (Hamilton, 2020; Corra, 2022). Immigrants from African countries like Kenya, Nigeria, and South Africa, for example, are shown to fare better in the U.S. labor market than those from countries like Somalia and Sudan.

## African immigrants compared with other immigrant groups

In comparing African immigrants with other immigrant groups, several points are also worth noting. First, recent analysis of U.S. Census data (Corra, 2022) shows that, like all immigrant groups, African immigrants exhibit varying levels of socioeconomic status that compare more and less favorably with differing groups and with differing measures. Second, as above, race is shown to be a salient, consistent, and statistically significant variable in labor market disparities. Yet, this finding is shown to be nuanced by multiple factors, including gender, region/country of origins and the specific variable being measured. Regional and country differences are shown to exist, with the salience of race being more pronounce among immigrants from Africa, Asia, and Europe. And the gendered nature of the effects of race is shown to be evident in the finding that Black women are shown to exhibit relatively favorable socioeconomic measures, relative to their White counterparts, and at least on some dimensions. Whereas Black men are shown to be consistently disadvantaged relative to their White counterparts.

## African immigrants in comparison with natives

As above, several points are notable here. First, African immigrants, both Black and White, are shown to compare favorably with several native groups on several dimensions. For example, the average percent with a college degree or more for African immigrants (about 54% for males and about 38% for females) is shown to be noticeably higher than that for all native groups, including non-Hispanic Whites. In fact, with the single exception of native Asians, African immigrants, both male and female, are shown to hold the highest averages of educational attainment (Corra, 2022). Only native Asians are shown to hold educational measures that are at per with those held by African immigrants. And this is shown to be true for both males and females.

Recent surveys (see, for example, Anderson and Connor, 2018; Simmons, 2018) highlight the notable educational attainment of African immigrants, relative to others, both native and immigrant. The title of a 2018 Los Angeles Times piece, “African immigrants are more educated than most — including people born in U.S.”(Simmons, 2018) is pointedly direct.^12^

An Immigration Policy Center analysis released last year (2022) reports that 42% of sub-Saharan Africans ages 25 and over in the U.S. held a bachelor's degree or higher, compared with 33% of all foreign- and U.S.-born adults.^13^ That report highlights immigrants from Nigeria and South Africa as examples of African immigrant groups with some of the highest education, with 64and 58%, respectively, holding at least a bachelor's degree, followed by Cameroonians (52%), Kenyans (49%), and Ghanaians (42%).

Moreover, a 2018 New American Economy study, “Power of the Purse: How Sub-Saharan Africans Contribute to the U.S. Economy,” profiles the type of degrees held by these immigrants.^14^ It reports that 1 in 3 of undergraduate degrees held by African immigrants were focused on science, technology, engineering, and math — “training heavily in demand by today's employers” (p. 2).

In addition, the LA Times piece noted above indicated that African immigrants are significantly more likely to have graduate degrees. And that a total of 16% of African immigrants then had a master's degree, medical degree, law degree or a doctorate, compared with only 11% of the U.S.-born population.

Yet, another key recent finding is the salience of race that is mediated by gender, wherein the effects of race are acutely salient among men, and less so among women, or sometimes take the opposite effect. For example, recent analysis of U.S. Census data (Corra, 2022) shows that, unlike their male counterparts, Black African immigrant women's net earnings are significantly higher than all groups of females, including native born non-Hispanic white women's. Indeed, very few groups are shown to out-earn Black African immigrant women. In short, the U.S. labor market tends to favor Black African women than black African men.

Finally, a key variable that has been recently noted is the role of migration itself (both internal and international) in producing labor market disparities (Butcher, 1994; Model, 2008; Hamilton, 2014, 2019, 2020). Here, the critique is that, unlike the native-born, immigrants are not from a sample of randomly selected individuals from their countries of origins. Rather, they are a highly selected group that systematically differ in some way from the larger population from which they emigrated. Hence, as economist Butcher (1994) suggests, such immigrants are appropriately comparable to native-born “internal movers” (frequently operationalized as the native-born living in states different from their state of birth) (see Butcher, 1994; Tolnay, 2003; Model, 2008; Hamilton, 2014, 2019, 2020).

Here, findings from a recent study by Hamilton (2014) are worth noting. Evaluating whether arrival cohorts of black immigrants from four sending regions–the English-speaking Caribbean, Latin America, Haiti, and Africa—converge with the earnings of four subgroups of U.S.-born men: native blacks taken together, native black movers, native black non-movers, and U.S.-born non-Hispanic whites, Hamilton offered results that are of critical import. First, he found lower weekly earnings for all cohorts of black immigrants upon arrival in the United States, relative to both native Black groups (blacks taken together, and native black movers) (Corra, 2022). Second, with respect to projections of convergence/divergence, Hamilton reported that: “Although the rate of earnings growth varies by birthplace, several arrival cohorts of black immigrants from English-speaking countries in Africa and the Caribbean are projected to overtake the earnings of native blacks (collectively) as their tenure of U.S. residence increases. Fewer of these arrival cohorts, however, are projected to converge with or surpass the earnings of native black movers, and no arrival cohort is projected to achieve earnings parity with native whites” (p. 977).

Moreover, Hamilton's (2014) study also included additional findings that are worth noting here. A key variable of distinction, for example, is linguistic heritage, i.e., immigrants from English and non-English speaking countries. He reports that: “the earnings of most arrival cohorts of immigrants from the English-speaking Caribbean, after residing in the United States for more than 20 years, are projected to converge with or slightly overtake those of U.S.-born black internal migrants. The findings also show three arrival cohorts of black immigrants from English-speaking African countries are projected to surpass the earnings of U.S.-born black internal migrants.”

Corra (2022) recent analysis, by contrast, shows that disparities among native Blacks are accentuated by internal migration/non-migration. Black movers are shown to generally exhibit measures that are more favorable than non-movers. Yet, in contrast to previous studies (Butcher, 1994; Model, 2008; Hamilton, 2014, 2019, 2020), the distinction between native internal migrants and non-migrants did not eliminate statistically significant variations in estimates favoring African immigrants, relative to native-born blacks.

## Discussion and conclusion

The foregoing review sought to summarize recent findings on the growth of African immigration to the United States in the past several decades. Several emerging patterns were noted, including the increasing diversity, yet also racialized portrait of this group. Key patterns of immigration shown include the shifting racial and gender composition of immigrants, as well as rising immigration from a wider range of African countries. Direct implications of these demographic shifts and their impacts on the foreign-born and U.S. Black populations were noted.

For future research, one clear evident implication of the shifting racial and gender composition of the African immigrant population is that they are not a monolithic group. Increasingly African immigrants are coming from countries and regions that are culturally, linguistically, politically and/or economically distinct.

It follows that a key conclusion from this review is that lumping African immigrants into one homogeneous group for theoretical and/or empirical analyses is problematic, to say the least. Hence, analyses of labor market disparities among African immigrants (or in comparison to other immigrants and/or natives) should clearly consider the emerging immense diversity among this group of immigrants.

More specifically, patterns reviewed in this paper suggest that research should pay close attention to the increasing diversity in the racial, ethnic and gender composition of the African immigrant population in the United States. For example, early migration theory and research (Chiswick, 1978; Carliner, 1980) portrayed a rather linear picture of U.S. immigration and successful immigrant adaptation. After a relatively short adjustment period, immigrants are said to “catch up” and/or “overtake” comparable natives in socioeconomic attainment. Chiswick (1978) estimated the “overtaking” point for immigrants at 10–15 years after immigration.

Recent theoretical approaches to the study of immigration, however, suggest that the continued multi-colored nature of U.S. society along ethnoracial lines means that immigrants experience a segmented form of assimilation. Some recent approaches to the study of migration, for example, emphasize the “context of reception” to the host society and the modes of incorporation of different groups into its labor market (Portes and Böröcz, 1989; Portes and Rumbaut, 2001, 2006). Here, one key variable is said to be racial status (Borch and Corra, 2010; Thomas, 2014; Sáenz and Manges Douglas, 2015; Corra, 2022). And in terms of migration, race comes to the forefront when the question focuses specifically on how racial status differentially influences immigrant adaptation (Dodoo and Takyi, 2002; Borch and Corra, 2010; Thomas, 2014; Corra, 2022). Again, according to Dodoo and Takyi (2002): “The condition of Africans in the diaspora proffers insight into not just their adaptation to their new countries, but also the nature of racial stratification at their destinations” (p. 913).

Finally, the notable variations in legal admission status shown to be associated with rising immigration from a wider range of African countries means research should also pay closer attention to how different groups of immigrants obtain legal admission status. Several questions come to mind, including: Are African immigrants more or less likely than other immigrant groups to be admitted as political or family-based immigrants? Alternatively, are African immigrants more or less likely than other immigrant groups to be admitted based on employment and skills-based preferences? How about African immigrants themselves, are some groups more or less likely to be admitted into the U.S. based on one entry status or the other? What socioeconomic inferences can be drawn from these?

These questions are important for both theoretical and practical reasons. For example, migration theory and research suggest that how well immigrants do may be uniquely tied to whether they are “economic” or “political” migrants. At least since Everett Lee's (1966) “A Theory of Migration,” researchers have hypothesized that “Politically motivated emigrants” are less positively selected (pushed) than “economically motivated emigrants (pulled) (Chiswick, 1978, 1999; Jasso and Rosenzweig, 1990a,b; Borjas, 1994; Tesfai, 2019). If many recent African immigrants have political rather than economic motives to relocate (Gordon, 1998), then one important proposition is that these immigrants will be less selective than other immigrant groups. Conversely, if some groups of African immigrants are more likely than others to be political rather than economic immigrants (Gordon, 1998; Corra, 2022), then another hypothesis is that the former will be less selective than the latter.^15^ More specifically, the type of visa an immigrant holds is hypothesized to influence immigrant incorporation (Jasso et al., 2000; Jasso, 2004; Corra, 2022).

## Author contributions

The author confirms being the sole contributor of this work and has approved it for publication.

## Footnotes

^1^As indicators of immigration flows, a key limitation of these figures is that, as official enumerations of legal permanent residence status, they are not inclusive of illegal/undocumented immigrants. Moreover, the reported year of admission is the year in which the individual immigrant is officially granted legal permanent residency. Increasingly, this is not the year in which the immigrant initially arrived (Model, 2008). For example, some “aliens already living in the United States, including certain undocumented immigrants, temporary workers, foreign students, and refugees, file an application for adjustment of status (to legal permanent residence) with I.N.S.” (United States Immigration Naturalization Service, 1998, p. 14).

^2^A reviewer of this paper rightfully asks for caution in the use of “voluntary” to characterize recent African immigration to the United States. That reviewer accurately noted that it is indicated in the text of this manuscript that: “…Figure 1 shows “voluntary” immigration from Africa. However, the footnote that describes these data...rightfully indicates that they could contain refugees who filed for an adjustment to their status.” The reviewer's point here is well-taken. Two additional notes of clarification, however, are worth making. First, as a point of clarification, my use of “voluntary” here is to make a distinction between Forced U.S. African immigration as enslaved, and all other forms of immigration. Arguably the former is qualitatively and distinctly unique. Yet, the need for clarification is clearly warranted–immigration as a refugee is indeed not voluntary. Second, size and temporal trends also matter. Corra's recent analysis of data from the U.S. Department of Homeland Security's Yearbook of Immigration Statistics, for example, shows that, of the 2,045,199 Africans granted legal permanent residency between the years 1996–2019, 801,876 (about 39.32%) were admitted as “Immediate relatives of U.S. citizens,” 160,888 (about 7.87%) were based on “Family-sponsored preferences,” 427,920 (about 20.92%) were admitted based on the Department of States' “Diversity lottery program,” and 124,967 (about 6.11%) were admitted based on employment-based preferences. Collectively, these four categories of admissions constituted about seventy-four percent (74.22%) of the total number of Africans granted legal permanent residency in the United States between 1996 and 2019. By contrast, 520,527 (about 25.50%) were admitted as Refugees and asylees. Notably, though in total this constitutes a sizeable percentage, Corra's trend analysis shows African immigration based on Refugee and Asylee status to be a rising, but very recent phenomenon. Moreover, such immigration is also shown to be less wide-spread across countries. In a 2022 Pew Research Center report, for example, Tamir and Anderson note that immigration from Somalia to the United States has been on the rise, resulting in a 205% increase in the Somali-born population in the U.S. since 2000. Immigration data from the U.S. Department of Homeland Security recently reviewed by Corra (2022; see below) shows the country of Somalia also has one of the highest percentages of immigrants granted permanent residency status as refugees and asylees.

^3^It is important to note here that, because they exclude undocumented immigrants, the data presented in Figure 1 does not completely reflect immigration patterns from Africa. A recent estimate is that, as of 2019, approximately 261,000 (2 percent) of the 11 million unauthorized immigrants in the United States were from sub-Saharan Africa (Lorenzi and Batalova, 2022). The data presented in Figure 1, however, does reflect “legal” or “documented” immigration patterns and trends, and thus provide useful estimations of immigration flows from Africa.

^4^This is in reference to top ten sending countries in any given time period, which has changed slightly between periods. Somalia and Sierra Leone, for example, did not reach the threshold of being one of the top ten countries representative of African immigrants in the United States until 2000. Similarly, Cameroon did not reach this threshold until 2010, but it remained as one of the top ten countries represented in the U.S. African immigrant population in 2019.

^5^A note of qualification here is that immigrants are not from a sample of randomly selected individuals from their countries of origins. Rather, they are a highly selected group that systematically differ in some way from the larger population from which they emigrated. Hence, a general hypothesis can be made that all categories of immigrants are selective, at least relative to natives (Butcher, 1994; Model, 2008; Hamilton, 2014, 2019, 2020).

^6^Funded by the National Science Foundation, the University of Minnesota, and the National Institutes of Health, the Integrated Public Use Microdata Series (IPUMS) are nationally representative samples of U.S. Census data specifically compiled and made available for social and economic research. Compiled and put together by the Minnesota Population Center, the series contains microdata samples (5%, 1%, etc.) of decennial U.S. Census as far back as 1850 and up to the 2010 decennial Census. And, as of the writing of this manuscript, it also currently has samples of the American Community Survey (ACS) from 2000 all the way to 2021. Conducted annually by the U.S. Census Bureau, the ACS is an annual statistical survey of a small (nationally representative) percentage of the population. For a complete description of the IPUMS dataset (including sample and variable descriptions, data compilation and storage), see the IPUMS website at http://www.ipums.org.

^7^I am indebted to a reviewer of this manuscript for bringing this important point to my attention. That reviewer observed that: “The recent decline in White African immigration, at a time of increasing North African immigration to the U.S. pointed by the author, is therefore significant.” The reviewer further suggested a possibility: “Could this be that these North Africans include Arabs, from countries such as Egypt, who are now refusing to identify as white?” Though beyond the scope of this paper, this is a distinct possibility (but see the paragraph to immediately follow). Also beyond the scope of this review, another possible explanation is an increasing number of “Black” immigrants from these countries.

^8^The U.S. Census Bureau defines the foreign born as individuals who had no U.S. citizenship at birth. African immigrants included those who recorded their birthplace in any of the African countries in the census documents. Of these, those who self-identified as “Black” are included in the Black foreign-born.

^9^Census data indicates that as of 2010, 74.3% of African immigrants were black, while 20% were white and 2.7% were Asian.

^10^See, for example Scroggins (1989), Model (1991, 1995, 2008), Dodoo (1991a,b,c,d); Dodoo (1997), Butcher (1994), Kalmijn (1996), Dodoo and Takyi (2002), Corra and Kimuna (2009), Borch and Corra (2010), Corra and Borch (2014), Hamilton (2014, 2019, 2020), Elo et al. (2015), Kusow et al. (2016, 2018), Tesfai (2017a,b); Tesfai (2019), and Nawyn and Park (2019).

^11^See, for example Oaxaca (1973), Beck et al. (1980), Hodson and Kaufman (1982), Coverman (1986), Reskin (1988), Williams (1992), Tomaskovic-Devey (1993), Budig and England (2001), Kaufman (2002), Cohen and Huffman (2003), Mishel et al. (2007), and England (2010).

^12^The article is accessible at: https://www.latimes.com/world/africa/la-fg-global-african-immigrants-explainer-20180112-story.html.

^13^The report is accessible at: https://www.migrationpolicy.org/article/sub-saharan-african-immigrants-united-states#English_Proficiency.

^14^The report is accessible at: https://research.newamericaneconomy.org/wp-content/uploads/sites/2/2018/01/NAE_African_V6.pdf.

^15^Another distinction between political and economic migrants is Portes and Rumbaut's expectation that political migrants (such as refugees) get various kinds of assistance from the government that economically motivated migrants do not get (Model, personal communication). One of the characteristics of “contexts of reception,” that expectation may or may not be applicable to African immigrants. Arguably, this expectation might apply primarily to Cubans. The forthcoming book, Seeking Refuge, Finding Inequality: Refugees Navigating Their Way, by Annette Lareau and Blair Sackett (also see Sackett and Lareau, 2022, Forthcoming), is an ethnographic study that documents the impediments that refugees to the US from the Democratic Republic of Congo encountered, trying to secure the benefits to which they were entitled.
